# Effect of Comorbidity Burden and Polypharmacy on Poor Functional Outcome in Acute Ischemic Stroke

**DOI:** 10.1007/s00062-022-01193-8

**Published:** 2022-07-13

**Authors:** Ewgenia Barow, Ann-Cathrin Probst, Hans Pinnschmidt, Marlene Heinze, Märit Jensen, David Leander Rimmele, Fabian Flottmann, Gabriel Broocks, Jens Fiehler, Christian Gerloff, Götz Thomalla

**Affiliations:** 1 Klinik und Poliklinik für Neurologie, Kopf- und Neurozentrum, University Medical Center Hamburg-Eppendorf, Martinistraße 52, 20246 Hamburg, Germany; 2 Institut für Medizinische Biometrie und Epidemiologie, University Medical Center Hamburg-Eppendorf, Hamburg, Germany; 3 Department of Diagnostic and Interventional Neuroradiology, University Medical Center Hamburg-Eppendorf, Hamburg, Germany

**Keywords:** Large vessel occlusion, Clinical outome, Charlson Comorbidity Index, Thrombectomy, Acute stroke

## Abstract

**Purpose:**

Comorbidities and polypharmacy are risk factors for worse outcome in stroke. However, comorbidities and polypharmacy are mostly studied separately with various approaches to assess them. We aimed to analyze the impact of comorbidity burden and polypharmacy on functional outcome in acute ischemic stroke (AIS) patients undergoing mechanical thrombectomy (MT).

**Methods:**

Acute ischemic stroke patients with large vessel occlusion (LVO) treated with MT from a prospective observational study were analyzed. Relevant comorbidity burden was defined as a Charlson Comorbidity Index (CCI) score ≥ 2, polypharmacy as the intake of ≥ 5 medications at time of stroke onset. Favorable outcome was a score of 0–2 on the modified Rankin scale at 90 days after stroke. The effect of comorbidity burden and polypharmacy on favorable outcome was studied via multivariable regression analysis.

**Results:**

Of 903 patients enrolled, 703 AIS patients (mean age 73.4 years, 54.9% female) with anterior circulation LVO were included. A CCI ≥ 2 was present in 226 (32.1%) patients, polypharmacy in 315 (44.8%) patients. Favorable outcome was less frequently achieved in patients with a CCI ≥ 2 (47, 20.8% vs. 172, 36.1%, *p* < 0.001), and in patients with polypharmacy (69, 21.9% vs. 150, 38.7%, *p* < 0.001). In multivariable regression analysis including clinical covariates, a CCI ≥ 2 was associated with lower odds of favorable outcome (odds ratio, OR 0.52, 95% confidence interval, 95% CI 0.33–0.82, *p* = 0.005), while polypharmacy was not (OR 0.81, 95% CI 0.52–1.27, *p* = 0.362).

**Conclusion:**

Relevant comorbidity burden and polypharmacy are common in AIS patients with LVO, with comorbidity burden being a risk factor for poor outcome.

**Electronic supplementary material:**

The online version of this article (10.1007/s00062-022-01193-8) contains supplementary material, which is available to authorized users.

## Introduction

Stroke is one of the leading causes of death and disability worldwide, thus constituting a challenge to the healthcare system [1]. Most strokes occur in older people with usually multiple comorbidities [2, 3]. Up to 90% of stroke patients have one or more comorbidities [4]. Comorbidities have been reported as risk and prognostic factors that pose a threat to survival and recovery after stroke [5–7]. Patients with a higher number of comorbidities show an increased risk of in-hospital mortality, independent of stroke severity [8], as well as 1 year mortality [5, 9]. Among stroke survivors, those with more comorbidities are more likely to have a poor functional outcome [5, 9], and to suffer from long-term complications [10].

Polypharmacy is a consequence of the ageing multimorbid population, with up to 57% of patients aged 65 years old or more being prescribed 5 or more medications [11, 12]. The highest level of prescribing is observed in patients with cardiovascular risk factors [13], especially diabetes and coronary heart disease [14]. In stroke patients polypharmacy is associated with worse functional outcome [15]. Furthermore, the intake of a higher number of drugs is associated with lower possibility of discharge home [16], with the severity of poststroke fatigue [17], progression of cognitive impairment [18], and increased number of falls [19].

However, data on the association between comorbidities and polypharmacy with stroke outcome is scarce and the approaches to assess comorbidities and polypharmacy vary substantially among available studies. Moreover, comorbidities and polypharmacy, which mutually determine each other, are often analyzed separately. The aim of the present study was to analyze the impact of comorbidities and polypharmacy on functional outcome in patients with severe stroke, i.e., acute ischemic stroke (AIS) patients with large vessel occlusion (LVO) treated with mechanical thrombectomy (MT).

## Methods

### Study Cohort

Consecutive AIS patients undergoing MT due to LVO, were enrolled in a prospective observational study between June 2015 and September 2020. Study protocols and procedures conformed to the Declaration of Helsinki and were approved by the local ethics committee. Informed consent was provided by patients or their legal representatives or waived, when patients lacked the capacity to give consent with no available proxy, or the patients died before consent could be obtained. Decisions on the appropriate treatment were made in an interdisciplinary team of neurologists and interventional neuroradiologists on a case-by-case basis. If appropriate, intravenous thrombolysis (IVT) was applied before MT, according to national guidelines.

Demographic characteristics, medical history including cardiovascular risk factors and other comorbidities, as well as medication history were recorded. Further information on stroke etiology, whether the patient was admitted directly to our center (mothership) or was referred from an external hospital (ship), time from symptom onset to groin puncture, time from symptom onset to recanalization, and successful reperfusion defined by a score of 2b or 3 on the Thrombolysis in Cerebral Infarction (TICI) scale on the final angiographic series were assessed. Favorable outcome was defined as modified Rankin Scale (mRS) score of 0–2 at 90 days after stroke indicating functional independence.

### Comorbidities and Polypharmacy

Pre-existing comorbidities were assessed and summarized according to the Charlson Comorbidity Index (CCI), which has been adjusted for ischemic stroke patients [5]. The CCI was developed to predict mortality on the basis of International Classification of Diseases, Ninth Revision, Clinical Modification codes [20]. The adjusted CCI comprises 16 diseases, which are assigned a score according to their disease severity. Finally, the sum of all scores provides a patient’s total score. Atrial fibrillation, arterial hypertension and dyslipidemia are not contained in the CCI and were therefore noted additionally. Atrial fibrillation was considered existent if either previously reported or detected on admission or during the hospital stay.

According to the most common definition, polypharmacy was defined as the intake of five or more medications at the time of stroke onset [21]. On-demand medication was not considered.

### Statistical Analysis

Clinical and demographic characteristics were compared between patients with and without a relevant comorbidity burden and polypharmacy, using Mann-Whitney U‑tests, χ^2^-tests, or Fisher’s exact tests as appropriate. The CCI score was dichotomized according to the prevalence of comorbidities with a cut-off of ≥ 2 points, standing for relevant comorbidity burden, as it has been suggested previously in the context of stroke outcome to define high comorbidity load [5]. Clinical variables were described by mean and standard deviation, median and interquartile ranges and with frequency and percentages, whichever is appropriate. Univariable and multivariable logistic regression analyses were done to test the effects of the independent variables age, stroke severity (National Institues of Health Stroke Scale, NIHSS), Alberta Stroke Program Early CT Score (ASPECTS), male sex, disability before admission (mRS before admission > 2), IVT, polypharmacy, relevant comorbidity burden (CCI score ≥ 2), arterial hypertension, atrial fibrillation and dyslipidemia, successful recanalization (TICI 2b/3), referral from an external hospital (ship) and prestroke living status on favorable outcome (mRS 0–2). In the multivariable logistic regression analyses, all independent variables were first entered in the regression equation (initial “full” model). Non-significant independent variables were then removed from the equation, following a stepwise-backward procedure (final model). Odds ratios with 95% confidence intervals and *p* values are reported. Effects of comorbidity (CCI score) × age interactions were also tested, considering the CCI score as a dichotomous (CCI ≥ 2 vs. CCI < 2) as well as continuous variable. Collinearity among independent variables was assessed based on the variance inflation factors (VIFs) of all independent variables involved in the logistic regression models. The level of significance was *p* < 0.05, two-sided. All statistics were done using International Business Machines Corporation (IBM) SPSS statistics version 28.0 (IBM Corporation, Armonk, NY, USA 2021).

## Results

### Patient Characteristics

**Fig. 1:**
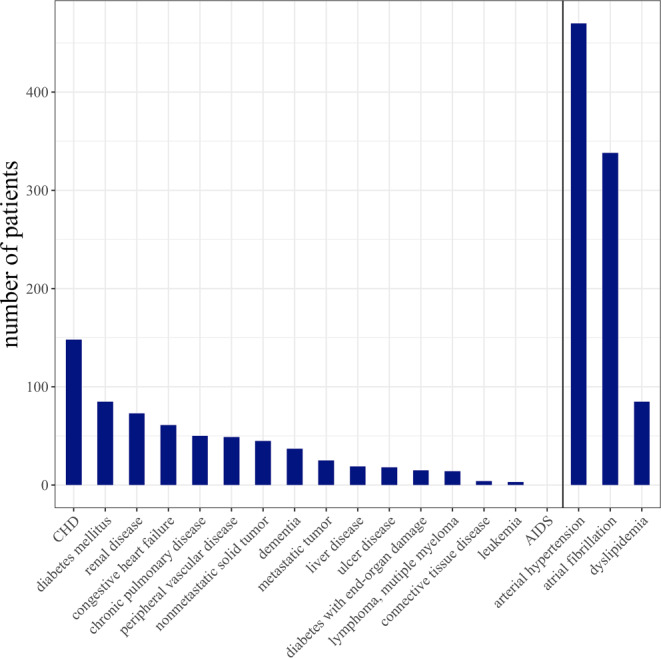
Distribution of individual diseases. The most prevalent comorbidities comprised by the Charlson Comorbidity Index (CCI) were coronary heart disease in 148 (21.1%) patients, diabetes in 85 (12.1%) patients and renal diseases in 73 (10.4%) patients (all left of the *vertical line*). Not captured by the CCI (right of the *vertical line*) were arterial hypertension, atrial fibrillation and dyslipidemia, present in 470 (66.9%), 338 (48.1%) and 84 (12.1%) patients. *CHD* Coronary Heart Disease, *AIDS* Acquired Immune Deficiency Syndrome

Of 903 acute ischemic stroke patients undergoing thrombectomy due to LVO 807 patients had available data on medication intake and comorbidities on admission as well as functional outcome (mRS) 90 days after stroke. Of these, 104 patients had a stroke with posterior circulation LVO and were therefore studied separately (see supplementary material for detailed information). The remaining 703 patients with a stroke in the anterior circulation were evaluated and included in the main analysis. Mean age was 73.4 years (standard deviation, SD, 12.7), 386 (54.9%) were female. All clinical characteristics are shown in Table 1. Almost one third of the patients (226, 32.1%) had a relevant comorbidity burden, defined by a CCI ≥ 2. The most prevalent comorbidities recorded by the CCI were coronary heart disease (in 148, 21.1% patients), followed by diabetes mellitus (in 85, 12.1% patients) and renal failure (in 73, 10.4% patients). Further comorbidities not captured by the CCI were arterial hypertension, present in 470 (66.9%) patients, atrial fibrillation (338, 48.1%), and dyslipidemia (84, 12.1%). The distribution of individual diseases is represented in Fig. 1.

**Table 1:** Baseline characteristics of all stroke patients with anterior circulation large vessel occlusion

Variable	All	CCI < 2	CCI ≥ 2	*p* value*	No polypharmacy	Polypharmacy	*p* value**
	*n* = 703 (100%)	*n* = 477 (67.9%)	*n* = 226 (32.1%)		*n* = 388 (55.2%)	*n* = 315 (44.8%)	
*Age, years mean (SD)*	73.4 (12.7)	72.5 (12.7)	75.5 (12.4)	**0.003**	71.2 (13.4)	76.2 (11.1)	**<** **0.001**
*Female, n (%)*	386 (54.9%)	268 (56.2%)	118 (52.2%)	0.364	211 (54.4%)	175 (55.6%)	0.814
*CCI score, median (IQR)*	1.00 (0.00–2.00)	N.A.	N.A.	N.A.	0.00 (0.00–1.00)	2.00 (1.00–3.00)	**<** **0.001**
*CCI score* *≥* *2, n (%)*	226 (32.1%)	N.A.	N.A.	N.A.	65 (16.8%)	161 (51.1%)	**<** **0.001**
*Arterial hypertension, n (%)*	470 (66.9%)	308 (64.6%)	162 (71.7%)	0.074	221 (57.0%)	249 (79.0%)	**<** **0.001**
*Atrial fibrillation, n (%)*	338 (48.1%)	208 (43.6%)	130 (57.5%)	**0.001**	154 (39.7%)	184 (58.4%)	**<** **0.001**
*Dyslipidemia, n (%)*	85 (12.1%)	43 (9.03%)	42 (18.6%)	**<** **0.001**	30 (7.75%)	55 (17.5%)	**<** **0.001**
*Medication on admission, mean (SD)*	4.49 (3.52)	3.45 (2.96)	6.69 (3.60)	**<** **0.001**	1.91 (1.56)	7.67 (2.53)	**<** **0.001**
*Polypharmacy, n (%)*	315 (44.8%)	154 (32.3%)	161 (71.2%)	**<** **0.001**	N.A.	N.A.	N.A.
*Independent living at home before stroke, n (%)*	627 (89.6%)	442 (92.9%)	185 (82.6%)	**<** **0.001**	364 (93.8%)	263 (84.3%)	**<** **0.001**
*Prestroke mRS score* *>* *2, n (%)*	70 (9.97%)	33 (6.92%)	37 (16.4%)	**<** **0.001**	23 (5.93%)	47 (15.0%)	**<** **0.001**
*Stroke etiology*	–	–	–	**<** **0.001**	–	–	**<** **0.001**
Cardioembolic stroke, *n* (%)	354 (50.4%)	219 (46.0%)	135 (59.7%)	–	165 (42.5%)	189 (60.2%)	–
Dissection, *n* (%)	11 (1.57%)	10 (2.10%)	1 (0.44%)	–	11 (2.84%)	0 (0.00%)	–
Atherosclerosis, *n* (%)	290 (41.3%)	216 (45.4%)	74 (32.7%)	–	183 (47.2%)	107 (34.1%)	–
Others, *n* (%)	26 (3.70%)	13 (2.73%)	13 (5.75%)	–	13 (3.35%)	13 (4.14%)	–
Stroke of undetermined etiology, *n* (%)	21 (2.99%)	18 (3.78%)	3 (1.33%)	–	16 (4.12%)	5 (1.59%)	–
*NIHSS score, median (IQR)*	15.0 (10.0–18.0)	15.0 (10.0–18.0)	15.0 (10.0–19.0)	0.574	15.0 (10.0–18.0)	15.0 (11.0–19.0)	0.335
*IVT, n (%)*	404 (57.5%)	302 (63.4%)	102 (45.1%)	**<** **0.001**	250 (64.6%)	154 (48.9%)	**<** **0.001**
*Ship, n (%)*	455 (64.7%)	318 (66.7%)	137 (60.6%)	0.138	253 (65.2%)	202 (64.1%)	0.827
*ASPECTS, median (IQR)*	8.00 (6.00–9.00)	8.00 (6.00–9.00)	8.00 (6.00–9.00)	0.158	8.00 (6.00–9.00)	8.00 (6.00–9.00)	0.526
*Time from symptom onset to groin puncture, median (IQR), min*	227 (155310)	230 (160–314)	225 (141–289)	0.372	230 (160–315)	225 (152–292)	0.639
*Time from symptom onset to recanalization, median (IQR), min*	275 (201–350)	284 (207–362)	265 (180–332)	0.181	284 (207–370)	273 (194–336)	0.410
*TICI 2b–3, n (%)*	526 (75.0%)	354 (74.4%)	172 (76.4%)	0.618	297 (76.7%)	229 (72.9%)	0.283
*mRS 90 days after stroke, median (IQR)*	4.00 (2.00–6.00)	4.00 (2.00–5.00)	5.00 (3.00–6.00)	**<** **0.001**	3.00 (1.00–5.00)	5.00 (3.00–6.00)	**<** **0.001**
*mRS 0–2 90 days after stroke, n (%)*	219 (31.2%)	172 (36.1%)	47 (20.8%)	**<** **0.001**	150 (38.7%)	69 (21.9%)	**<** **0.001**
*mRS 6 90 days after stroke, n (%)*	178 (25.3%)	86 (18.0%)	92 (40.7%)	**<** **0.001**	66 (17.0%)	112 (35.6%)	**<** **0.001**

*ASPECTS* Alberta Stroke Program Early CT Score, *CCI* Charlson Comorbidity Index, *IQR* interquartile range, *IVT* Intravenous Thrombolysis, *mRS* Modified Rankin Scale, *NIHSS* National Institutes of Health Stroke Scale, *SD* Standard Deviation, *TICI* Thrombolysis in Cerebral Infarction

*p values given for the comparison of patients with a relevant comorbidity burden and without

**p values given for the comparison of patients with polypharmacy and without

**Fig. 2:**
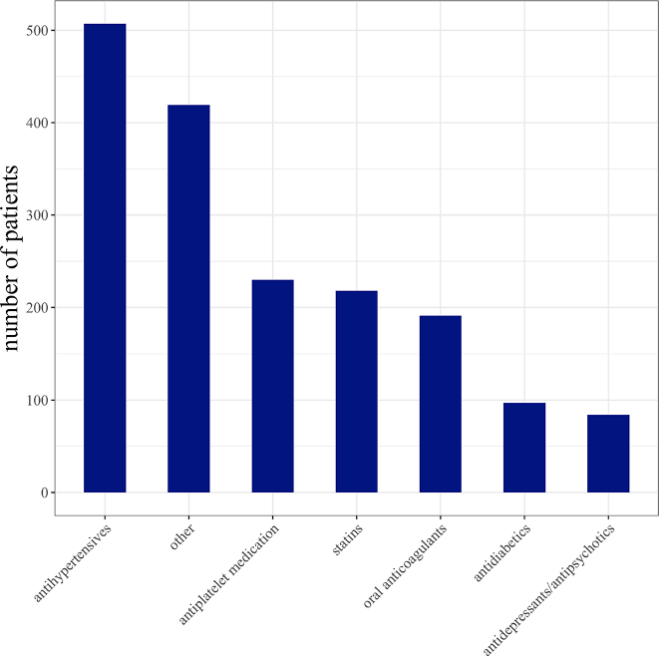
Number of patients taking at least one medication from different medication classes. 507 (72.1%) patients were taking at least one antihypertensive drug making it the most common type of medication, followed by antiplatelets (230 [32.7%] patients), statins (218 [31.9%] patients) and anticoagulants (191 [27.2%] patients), antidiabetics and antidepressants/antipsychotics

The patients were taking on average 4.5 (SD, 3.5) medications with 388 (55.2%) patients taking 5 or more medications. The proportion of the different medication groups is shown in Fig. 2. Antihypertensives were the most frequently taken medication (by 507, 72.1% patients), followed by antiplatelets (230, 32.7% patients), statins (218, 31.0 patients) and anticoagulants (191, 27.2% patients), all common medications in patients with cardiovascular diseases.

### Group Comparison by Comorbidity and Polypharmacy

Patients with a relevant comorbidity burden (CCI ≥ 2) were on average older, presented more often with pre-existing disability (higher prestroke mRS) and were less frequently independent in their living situation before admission, comparable to patients with polypharmacy (≥ 5 drugs, see Table 1). At 90 days after stroke both patients with relevant comorbidity burden and patients with polypharmacy, had poor functional outcome (mRS) in general, and additionally had higher mortality rates (mRS 6). For all baseline characteristics see Table 1.

### Influence of CCI Score and Polypharmacy on Favorable Outcome

Univariable regression analysis revealed a negative association of CCI ≥ 2 (odds ratio, OR 0.47, 95% confidence interval, 95%CI 0.32–0.68, *p* < 0.001), and polypharmacy (OR 0.44, 95%CI 0.32–0.62, *p* < 0.001) with favorable outcome. A stepwise-backward multivariable regression analysis revealed that a CCI ≥ 2 was significantly associated with an almost 50% reduced chance of favorable outcome (OR 0.52, 95%CI 0.33–0.82, *p* = 0.005), while polypharmacy had no significant effect. Nagelkerke’s R^2^ values for CCI were 0.029 (univariable regression analysis), 0.419 (multivariable regression analysis, full model) and 0.409 (multivariable regression analysis, final model), demonstrating that using the CCI as an ordinal or metric independent variable affected the performance of the model only marginally. The effect of comorbidities on outcome was also not modified by age, as age × CCI score interactions were not significant, whether the CCI score was used as a dichotomous or a continuous variable (*p* = 0.764 and *p* = 0.96, respectively). The variance inflation factors (VIFs) of all independent variables involved in the logistic regression models ranged between 1.01 and 1.6, indicating negligible collinearity. Univariable and multivariable analyses are presented in Table 2.

**Table 2:** Logistic regression analysis between variables and favorable outcome

Independent variable	Univariable (*n* = from 679 to 703)				Multivariable, full model (*n* = 673)				Multivariable, final model (stepwise-backward, *n* = 676)			
	OR	Lower 95% CI limit	Upper 95% CI limit	*p* value	OR	Lower 95% CI limit	Upper 95% CI limit	*p* value	OR	Lower 95% CI limit	Upper 95% CI limit	*p* value
CCI score ≥ 2	0.466	0.321	0.675	0.000	0.558	0.341	0.911	0.020	0.520	0.328	0.824	0.005
Polypharmacy	0.445	0.318	0.623	0.000	0.813	0.521	1.269	0.362	–	–	–	–
Arterial hypertension	0.601	0.431	0.838	0.003	1.166	0.746	1.823	0.500	–	–	–	–
Atrial fibrillation	0.629	0.455	0.869	0.005	1.127	0.731	1.739	0.588	–	–	–	–
Dyslipidemia	0.803	0.483	1.334	0.397	1.334	0.697	2.553	0.385	–	–	–	–
Age	0.949	0.936	0.962	0.000	0.939	0.921	0.957	0.000	0.940	0.924	0.957	0.000
Male sex	1.968	1.424	2.719	0.000	1.516	1.010	2.276	0.045	1.618	1.085	2.412	0.018
Prestroke mRS score > 2	0.056	0.014	0.232	0.000	0.117	0.023	0.593	0.010	0.064	0.014	0.291	0.000
Prestroke living status: Independent at home	–	–	–	0.000	–	–	–	0.189	–	–	–	–
Prestroke living status: nursing at home	0.106	0.014	0.797	0.029	0.384	0.045	3.295	0.383	–	–	–	–
Prestroke living status: nursing home	0.073	0.018	0.303	0.000	0.217	0.035	1.352	0.102	–	–	–	–
NIHSS score	0.923	0.897	0.950	0.000	0.927	0.896	0.959	0.000	0.927	0.896	0.959	0.000
Aspects	1.227	1.120	1.345	0.000	1.349	1.197	1.520	0.000	1.338	1.190	1.505	0.000
Ship	0.735	0.529	1.022	0.068	0.650	0.428	0.988	0.044	0.648	0.429	0.981	0.040
IVT	2.019	1.441	2.829	0.000	1.806	1.183	2.758	0.006	1.805	1.191	2.735	0.005
TICI 2b/3	4.131	2.554	6.679	0.000	5.520	3.177	9.588	0.000	5.393	3.118	9.328	0.000

*ASPECTS* Alberta Stroke Program Early CT Score, *CCI* Charlson Comorbidity Index, *CI* Confidence Interval, *IVT* Intravenous Thrombolysis, *mRS* Modified Rankin Scale, *NIHSS* National Institutes of Health Stroke Scale, *TICI* Thrombolysis in Cerebral Infarction

## Discussion

In the present analysis of a prospective observational cohort study, we analyzed the effect of comorbidities and polypharmacy on functional outcome in stroke patients with anterior circulation LVO undergoing MT. Relevant comorbidity burden was associated with poor functional outcome, while polypharmacy was not, when adjusting for important clinical covariates. Our study results extend previous findings by systematically studying the effect of comorbidities together with polypharmacy on functional outcome in a large sample of patients with severe stroke from clinical practice.

A relevant comorbidity burden was present in almost one third (32%) of our patients, which is in line with previous studies, reporting a prevalence in 29–65% of stroke patients [5, 9, 22–24]. Furthermore, a relevant comorbidity burden was associated with poor outcome, with a mortality rate of over 40% at 90 days after stroke, all in line with preceding investigations [5, 8, 9, 25].

When interpreting any study results on the effect of comorbidities on stroke outcome, study designs must be examined closely. Difficulties have arisen from previous studies assessing comorbidities by simply summing up the number of diseases, weighting them all equally. A standardized method to assess comorbidities has been established with the introduction of the CCI score [20]. Initially studied in a cohort of women with breast cancer, the CCI score aimed to predict mortality on the basis of International Classification of Diseases. The CCI score, adjusted for stroke patients, comprises 16 various diseases, which are assigned a weight according to their severity, summing up to an individual total score [5]. The cut-off of a CCI score ≥ 2 has been defined as a high comorbidity load in the context of stroke [5].

The CCI has certain limitations when used to study stroke cohorts, as it misses some of the most common comorbidities in this patient group: arterial hypertension, atrial fibrillation and dyslipidemia [26]. We have overcome this limitation of the CCI score by taking arterial hypertension, atrial fibrillation and dyslipidemia into account, which were highly prevalent in our patient cohort. Our study extends previous studies on comorbidities in stroke patients, which were mostly performed in small patient groups with different stroke etiologies, by providing a large sample size of severely affected stroke patients [8, 10, 23, 24, 27]. Moreover, we combined the analysis of comorbidities with the evaluation of polypharmacy.

Comorbidities can furthermore be associated with frailty, a syndrome also consisting of cognitive impairment and disability [28]. Frailty has been recently identified as a predictor for poor outcome in ischemic stroke patients [29, 30], and a recommendation to screen all patients over the age of 70 years for frailty has been made by an international expert consensus [28]. However, further investigations are needed, as not all comorbidities result in frailty, and a clear consensus on the best method to assess frailty in stroke patients has not been established yet. Polypharmacy is a phenomenon typical for an ageing population with multiple diseases and a highly developed medical system with widely adopted implementation of guideline recommendations. Our results confirmed a high prevalence of polypharmacy in stroke patients with up to 45% of our patients taking 5 or more medications [16, 17, 19]. Polypharmacy in stroke patients have been reported to be associated with poor functional outcome [15]. In accordance to that, poor functional outcome was also more common in our patients with polypharmacy. But in the multivariable regression analysis including other important clinical characteristics, a significant association between polypharmacy and outcome was not observed in our study. Possible explanations for the divergent results might be the higher age, the higher comorbidity burden and the pronounced stroke severity of our patients.

The current analysis has an important strength as the effect of a relevant comorbidity burden and polypharmacy on stroke outcome was studied in a real-world population. The consequences of real-world data for clinical practice have been demonstrated previously [31, 32]. The results of this study again underline the importance of real-world data outside the scope of randomized controlled trials (RCTs) with their strict inclusion and exclusion criteria. Several criteria applying to our cohort, such as higher age, relevant comorbidity burden and substantial prestroke disabilities, as well as referral from an external hospital for treatment would have led to an exclusion of a relevant number of patients from RCTs. Despite clear and necessary evidence provided by RCTs, real-world data are needed as they reflect everyday clinical practice across all patient groups. Furthermore, when especially considering our study results, real-world data help to discuss questions not sufficiently addressed in RCTs, such as if MT might be overperformed in a subpopulation of patients with an initially poor prognosis. Finally, real-world data are needed to generate hypotheses for future RCTs.

Our study has some limitations. As the analysis is based on observational registry data, findings have to be considered hypothesis-generating, and no causality can be assumed from the observed associations as to a potentially reduced benefit of thrombectomy in patients suffering from severe comorbidity. Our results might support prognostication of outcome after MT in patients with comorbidities, but no specific recommendations considering the appropriate treatment can be concluded. Neither adverse drug effects nor drug-drug interactions were considered, as we did not aim for a detailed investigation of individual drugs but rather for the effect of the overall load of medication taken at the time of stroke, as defined by polypharmacy. Predictors in the final multivariable regression analysis can therefore be representative for variables which are not included in the final regression analysis.

## Conclusion

Relevant comorbidity burden and polypharmacy are highly prevalent in acute stroke patients with anterior circulation LVO. A relevant comorbidity burden is a risk factor for worse functional outcome, independent of pre-existing polypharmacy, while polypharmacy is not.

## Supplementary Information



