# Supplementary material for: Effect of Comorbidity Burden and Polypharmacy on Poor Functional Outcome in Acute Ischemic Stroke
Source: Clin Neuroradiol. 2022 Jul 13;33(1):147–54. doi: 10.1007/s00062-022-01193-8 (PMC10014774; doi:10.1007/s00062-022-01193-8)

**Supplementary Table 1. Baseline characteristics of stroke patients with posterior circulation large vessel occlusion**

| **Variable** | **All** | **CCI<2** | **CCI≥2** | **p-Wert** | **Ohne Polypharmazie** | **Polypharmazie** | **p-Wert** |
| --- | --- | --- | --- | --- | --- | --- | --- |
|  | **N=104 (100%)** | **N=73 (70.2%)** | **N= 31 (29.8%)** |  | **N=61 (58.7%)** | **N= 43 (41.3%)** |  |
| Age, mean (SD) | 70.5 (12.8) | 70.0 (13.3) | 71.6 (11.6) | 0.549 | 68.1 (13.7) | 73.8 (10.5) | **0.019** |
| Female, n (%) | 44 (42.3%) | 25 (34.2%) | 19 (61.3%) | **0.019** | 29 (47.5%) | 15 (34.9%) | 0.278 |
| Independent living at home before stroke, n (%) | 94 (91.3%) | 66 (91.7%) | 28 (90.3%) | 0.372 | 57 (95.0%) | 37 (86.0%) | 0.190 |
| Prestroke mRS score, median (IQR) | 0.00 [0.00-0.00] | 0.00 [0.00-0.00] | 0.00 [0.00-1.00] | 0.029 | 0.00 [0.00-0.00] | 0.00 [0.00-1.00] | **0.017** |
| Prestroke mRS score 0-2, median (IQR) | 90 (86.5%) | 64 (87.7%) | 26 (83.9%) | 0.754 | 55 (90.2%) | 35 (81.4%) | 0.318 |
| Prestroke mRS score >2, n (%) | 14 (13.5%) | 9 (12.3%) | 5 (16.1%) | 0.754 | 6 (9.84%) | 8 (18.6%) | 0.318 |
| Stroke etiology |  |  |  | 0.234 |  |  | 0.531 |
| - Cardioembolic stroke, n (%) | 37 (35.6%) | 24 (32.9%) | 13 (41.9%) |  | 18 (29.5%) | 19 (44.2%) |  |
| - Dissection, n (%) | 2 (1.92%) | 1 (1.37%) | 1 (3.23%) |  | 2 (3.28%) | 0 (0.00%) |  |
| - Atherosclerosis , n (%) | 53 (51.0%) | 41 (56.2%) | 12 (38.7%) |  | 34 (55.7%) | 19 (44.2%) |  |
| - Others, n (%) | 5 (4.81%) | 4 (5.48%) | 1 (3.23%) |  | 3 (4.92%) | 2 (4.65%) |  |
| - Stroke of undetermined etiology, n (%) | 7 (6.73%) | 3 (4.11%) | 4 (12.9%) |  | 4 (6.56%) | 3 (6.98%) |  |
| CCI score, median (IQR) | 1.00 (0.00-2.00) | N.A. | N.A. | N.A. | 0.00 [0.00-1.00] | 1.00 [0.50-2.00] | **0.002** |
| CCI score ≥ 2, n (%) | 31 (29.8%) | N.A. | N.A. | N.A. | 12 (19.7%) | 19 (44.2%) | **0.013** |
| Medication on admission, mean (SD) | 4.23 (3.29) | 3.63 (3.08) | 5.65 (3.37) | **0.006** | 1.97 (1.51) | 7.44 (2.29) | <**0.001** |
| Polypharmacy, n (%) | 43 (41.3%) | 24 (32.9%) | 19 (61.3%) | **0.013** | N.A. | N.A. | N.A. |
| Arterial hypertension, n (%) | 84 (80.8%) | 57 (78.1%) | 27 (87.1%) | 0.427 | 45 (73.8%) | 39 (90.7%) | 0.057 |
| Dyslipidemia, n (%) | 12 (11.5%) | 11 (15.1%) | 1 (3.23%) | 0.103 | 4 (6.56%) | 8 (18.6%) | 0.069 |
| Atrial fibrillation, n (%) | 38 (36.5%) | 25 (34.2%) | 13 (41.9%) | 0.602 | 18 (29.5%) | 20 (46.5%) | 0.117 |
| NIHSS score, median (IQR) | 18.0 [8.00-42.0] | 16.0 [8.00-42.0] | 23.5 [11.0-42.0] | 0.117 | 14.0 [8.00-42.0] | 19.5 [9.25-42.0] | 0.408 |
| IVT, n (%) | 50 (48.1%) | 37 (50.7%) | 13 (41.9%) | 0.547 | 31 (50.8%) | 19 (44.2%) | 0.640 |
| Ship, n (%) | 62 (59.6%) | 43 (58.9%) | 19 (61.3%) | 0.993 | 39 (63.9%) | 23 (53.5%) | 0.386 |
| Time from symptom onset to groin puncture, median (IQR), min | 210 [150-315] | 227 [150-324] | 200 [160-295] | 0.828 | 237 [154-350] | 173 [150-295] | 0.344 |
| Time from symptom onset to recanalization, median (IQR), min | 288 [206-380] | 306 [207-363] | 263 [207-379] | 0.659 | 310 [223-400] | 266 [201-342] | 0.357 |
| TICI 2b-3, n (%) | 85 (82.5%) | 59 (80.8%) | 26 (86.7%) | 0.671 | 49 (81.7%) | 36 (83.7%) | 0.994 |
| mRS 90 days after stroke, median (IQR) | 4.00 [2.00-6.00] | 4.00 [2.00-6.00] | 4.00 [2.00-6.00] | 0.457 | 3.00 [2.00-5.00] | 5.00 [3.00-6.00] | **0.020** |
| mRS 0-2 90 days after stroke, n (%) | 32 (30.8%) | 23 (31.5%) | 9 (29.0%) | 0.986 | 23 (37.7%) | 9 (20.9%) | 0.107 |
| mRS 6 90 days after stroke, n (%) | 31 (29.8%) | 20 (27.4%) | 11 (35.5%) | 0.555 | 14 (23.0%) | 17 (39.5%) | 0.109 |

Abbreviations: CCI, Charlson Comorbidity Index; IQR, Interquartile Range; IVT, Intravenous Thrombolysis; mRS, Modified Rankin Scale; NIHSS, National Institutes of Health Stroke Scale; SD, Standard Deviation; TICI, Thrombolysis in Cerebral Infarction.

**Supplementary Table 2. Logistic regression analysis between variables and favourable outcome**

| **Independent variable** | **Univariable (n = from 103 to 104)** | | | | **Multivariable, full model (n = 101)** | | | | **Multivariable, final model (stepwise-backward, n = 101)** | | | |
| --- | --- | --- | --- | --- | --- | --- | --- | --- | --- | --- | --- | --- |
|  | **OR** | **lower 95% CI limit** | **upper 95% CI limit** | **p value** | **OR** | **lower 95% CI limit** | **upper 95% CI limit** | **p value** | **OR** | **lower 95% CI limit** | **upper 95% CI limit** | **p value** |
| Age | 0,972 | 0,941 | 1,004 | 0,087 | 0,991 | 0,941 | 1,043 | 0,722 |  |  |  |  |
| CCI score ≥ 2 | 0,889 | 0,355 | 2,230 | 0,803 | 1,699 | 0,458 | 6,310 | 0,428 |  |  |  |  |
| Arterial hypertension | 0,787 | 0,281 | 2,207 | 0,649 | 1,086 | 0,253 | 4,655 | 0,911 |  |  |  |  |
| Atrial fibrillation | 1,286 | 0,546 | 3,028 | 0,564 | 3,236 | 0,885 | 11,837 | 0,076 |  |  |  |  |
| Dyslipidemia | 0,179 | 0,022 | 1,450 | 0,107 | 0,223 | 0,021 | 2,410 | 0,216 |  |  |  |  |
| Pre-stroke living status: Independent at home |  |  |  | 1,000 |  |  |  | 1,000 |  |  |  |  |
| Pre-stroke living status: Nursing at home | 0,000 | 0,000 |  | 1,000 | 0,000 | 0,000 |  | 1,000 |  |  |  |  |
| Pre-stroke living status: Nursing home | 0,000 | 0,000 |  | 0,999 | 0,000 | 0,000 |  | 0,999 |  |  |  |  |
| MRS before admission > 2 | 0,146 | 0,018 | 1,172 | 0,070 | 0,751 | 0,056 | 10,067 | 0,829 |  |  |  |  |
| NIHSS score | 0,941 | 0,910 | 0,973 | 0,000 | 0,923 | 0,887 | 0,962 | 0,000 | 0,937 | 0,905 | 0,969 | 0,000 |
| Polypharmacy | 0,437 | 0,178 | 1,074 | 0,071 | 0,284 | 0,069 | 1,167 | 0,081 |  |  |  |  |
| Male sex | 1,105 | 0,474 | 2,574 | 0,817 | 2,624 | 0,720 | 9,561 | 0,144 |  |  |  |  |
| Ship | 1,443 | 0,607 | 3,431 | 0,406 | 0,891 | 0,275 | 2,885 | 0,847 |  |  |  |  |
| IVT | 1,339 | 0,581 | 3,087 | 0,493 | 2,293 | 0,708 | 7,432 | 0,166 |  |  |  |  |
| TICI 2b/3 | 4,364 | 0,939 | 20,269 | 0,060 | 6,573 | 1,148 | 37,628 | 0,034 | 6,461 | 1,295 | 32,236 | 0,023 |

Abbreviations: CCI, Charlson Comorbidity Index; CI, Confidence Interval; IVT, Intravenous Thrombolysis; mRS, Modified Rankin Scale; NIHSS, National Institutes of Health Stroke Scale; TICI, Thrombolysis in Cerebral Infarction.

**Supplementary Figure 1. Distribution of the Charlson Comorbidity Index (CCI) score**

Relevant comorbidity burden, defined by a CCI score ≥ 2 (see vertical line), was present in 31 (29.8%) patients.

**
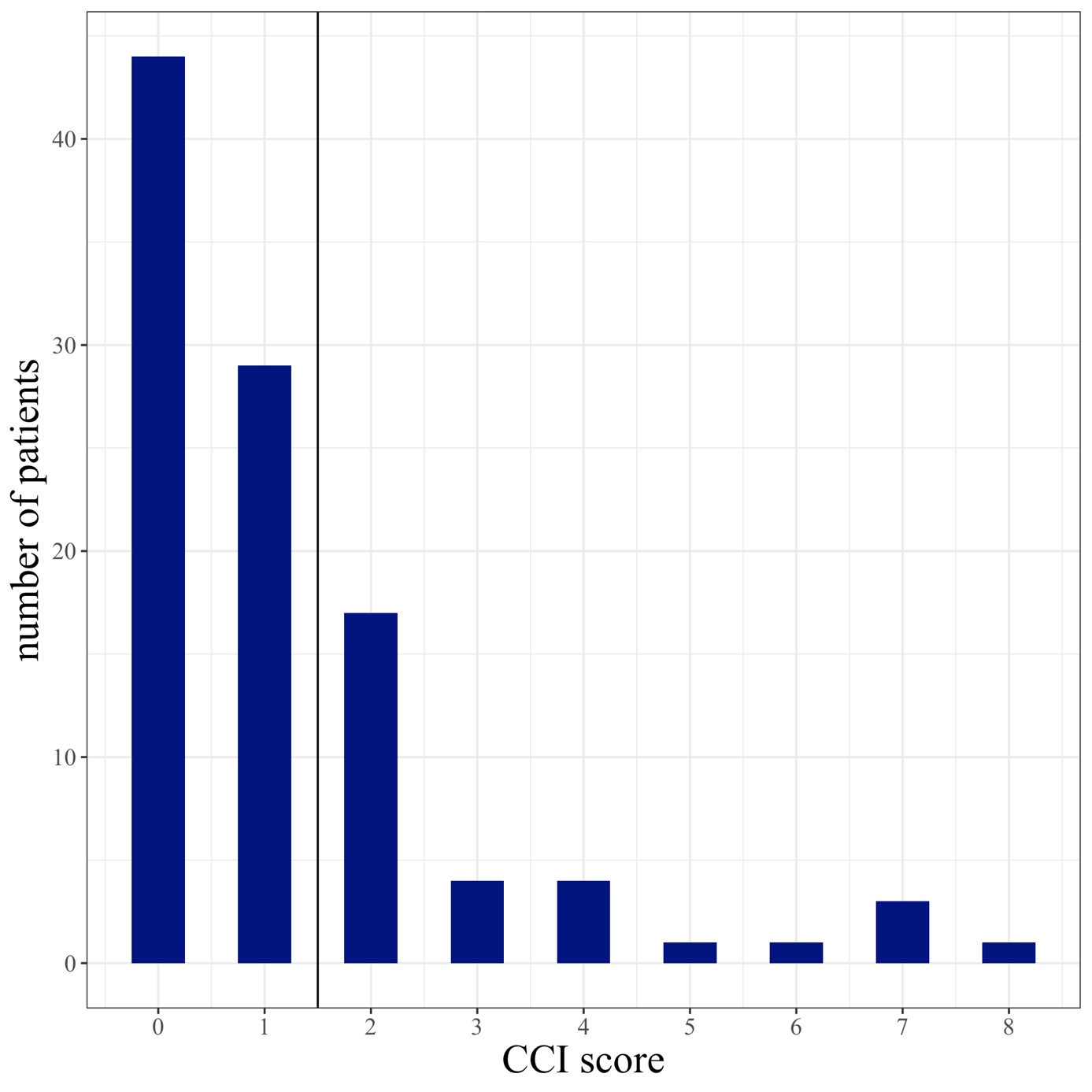
**

**Supplementary Figure 2. Distribution of individual diseases comprised in the Charlson Comorbidity Index (CCI)**

The most prevalent comorbidities were coronary heart disease in 23 (22.1%) patients, diabetes in 20 (19.2%) patients and congestive heart failure in 7 (6.7%).

Abbreviations: CHD, Coronary Heart Disease; AIDS, Acquired Immune Deficiency Syndrome.

**
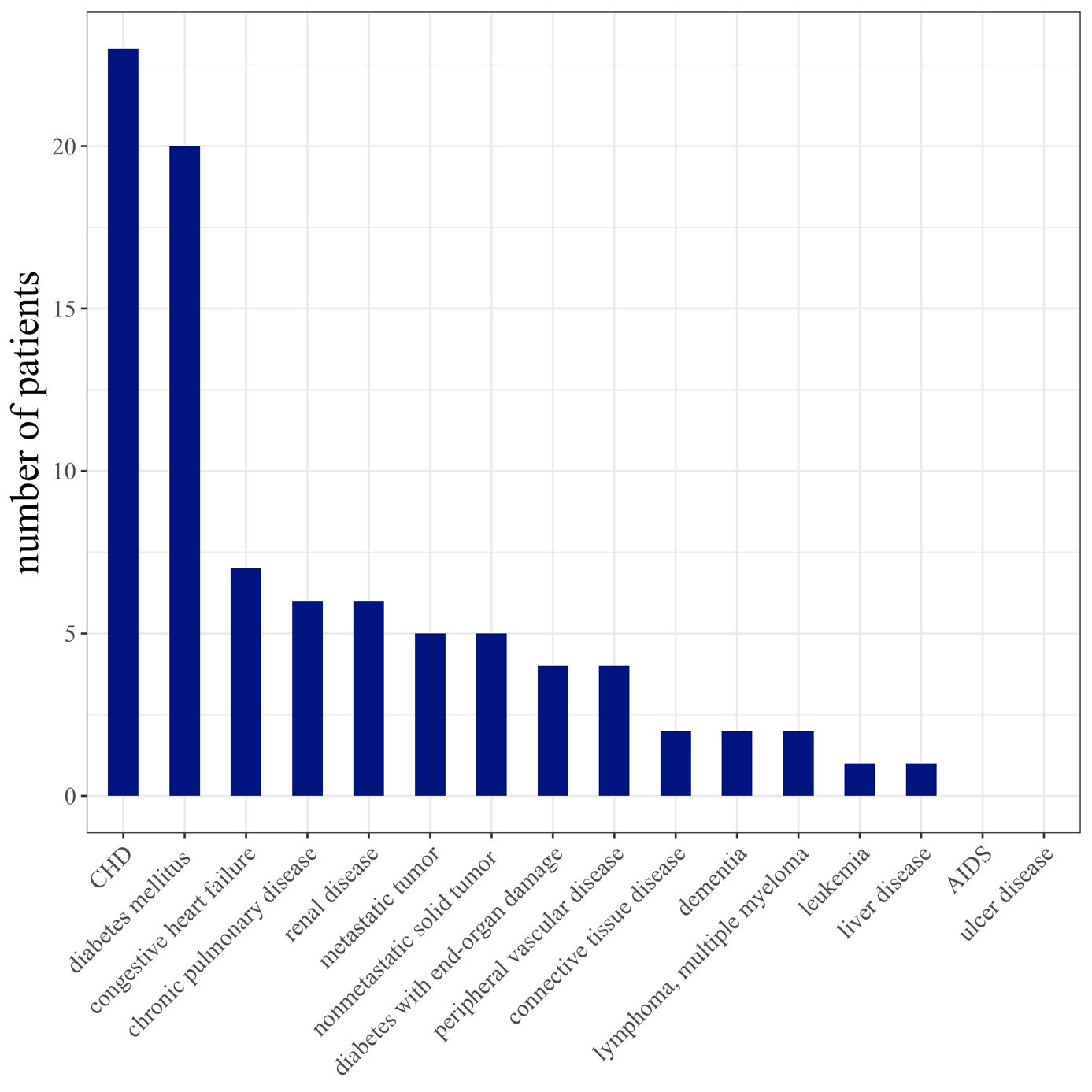
**

**Supplementary Figure 3. Distribution of medication intake**

The patients were taking on average 4.2 (3.3) medications. Polypharmacy was defined as an intake of 5 or more medications (see vertical line) and was observed in 43 (41.3%) patients.

**
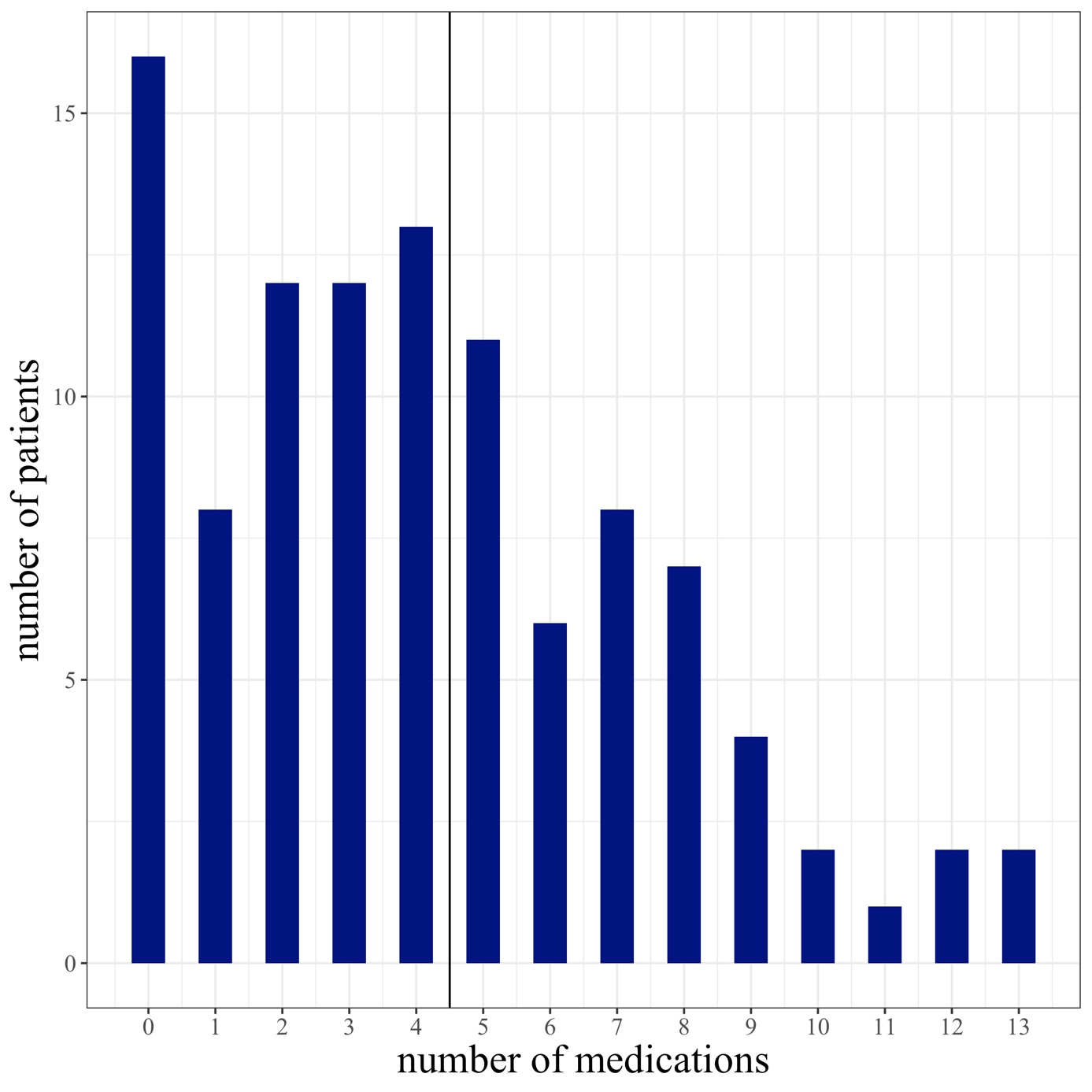
**

**Supplementary Figure 4. Number of patients taking at least one medication from different medication classes**

73 (70.2%) patients were taking at least one antihypertensive drug making it the most common type of medication, followed by antiplatelets (39 [37.5%] patients), statins (31 [29.8%]) and antidiabetics (21 [20.2%] patients), anticoagulants (14 [13.5%] patients) and antidepressants/antipsychotics (12 [11.5%] patients).


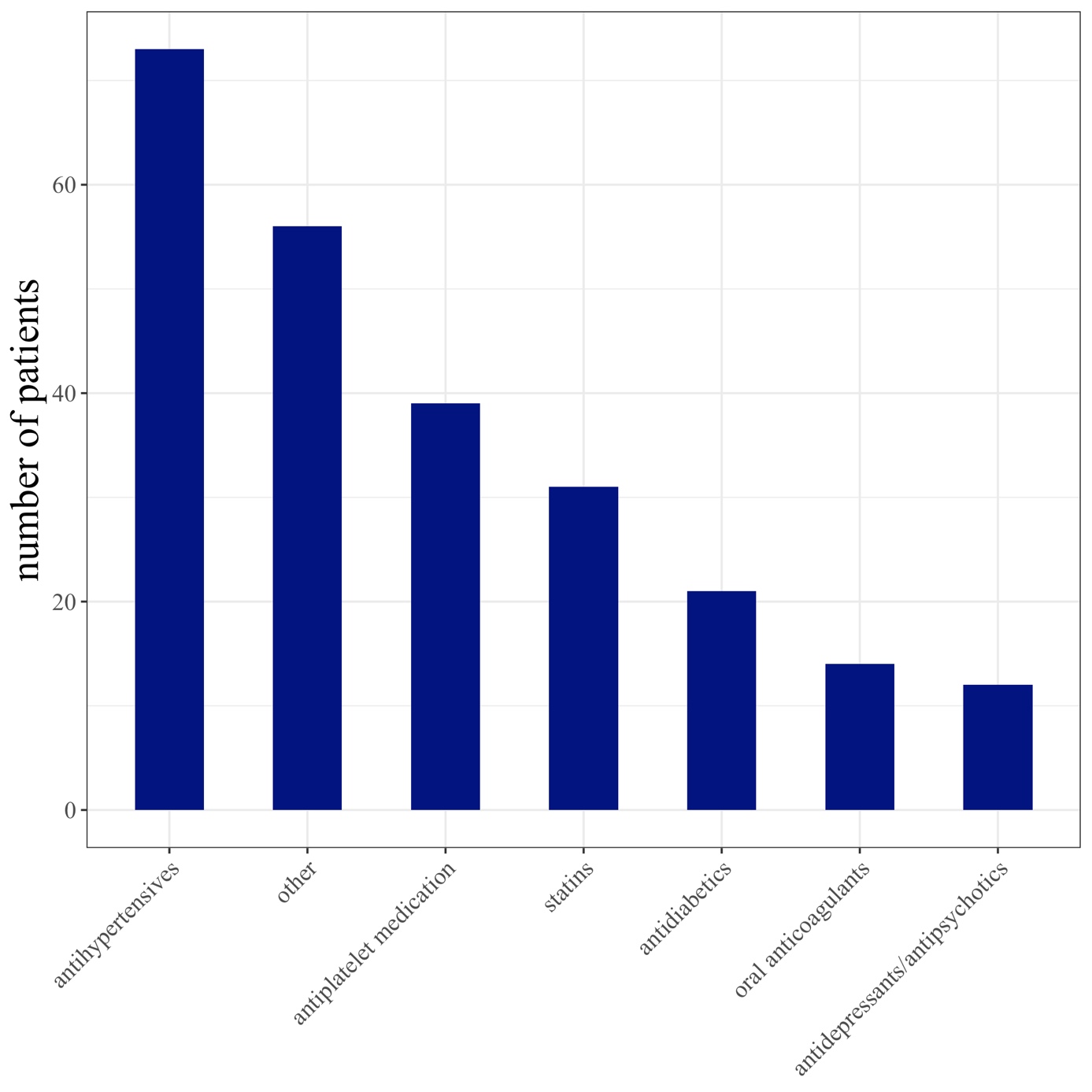

Supplement: Supplementary file 1 [file 62_2022_1193_MOESM1_ESM.docx]
